# Association of Aldosterone and Cortisol with Cardiovascular Risk Factors in Prehypertension Stage

**DOI:** 10.1155/2012/906327

**Published:** 2012-08-16

**Authors:** Sadiqa Badar Syed, Masood Anwar Qureshi

**Affiliations:** ^1^Department of Physiology, Medical and Dental College, Bahria University, Karachi 75260, Pakistan; ^2^Department of Physiology, Dow University of Health Sciences, Karachi 74200, Pakistan

## Abstract

*Background*. The Pakistani population has higher incidence of cardiovascular (CV) diseases at younger ages, due to undiagnosed, uncontrolled hypertension (HTN). A variety of associated HTN stressors is also reported. The study plans to understand the variables associated with initiation of HTN in this population. *Objective*. To find plasma aldosterone and cortisol relationship with some CV risk factors (obesity, dyslipidemia, hyperglycemia, sodium and potassium) in different stages of HTN particularly prehypertension. *Subjects and Methods*. The study conducted on 276 subjects (25–60 years), classified into prehypertensive (*n* = 55), HTN stage-1 (*n* = 70) and II (*n* = 76) according to 7th JNC report and compared with normotensive controls (*n* = 75). The anthropometric profiles (height, weight, waist circumference, Body Mass index) and BP recorded. Serum cortisol, aldosterone, total cholesterol, Low density lipoproteins, blood glucose, Na^+^ and K^+^, using standard laboratory techniques, were determined in fasting blood samples. *Results*. Subjects were mostly overweight and obese (80%, 90%, and 76% in pre-HTN, stage-I and II versus 69% in controls). The aldosterone level (ng/dl) was in higher normal range (9.17–12.41) and significantly correlated to BMI (0.587) in controls, and to TC (0.726) and LDL (0.620) in pre-HTN stage-I. The cortisol level was positively correlated (*P* < 0.01) to BMI (0.538), Na^+^ (0.690) and K^+^ (0.578) in control, and to BMI (0.628) and WC (0.679) in pre-HTN group, showing its association with BMI > 25. *Conclusion*. Pre-HTN stage among Pakistani population with successive increase in various risk factors of HTN in relation to aldosterone and cortisol has been identified. Interaction of the risk factors with endogenous levels of these hormones may initiate stages of HTN.

## 1. Introduction 

Hypertension (HTN) is a major risk factor for target organ damage leading to many major diseases such as myocardial infarction, stroke, impaired renal function, and ultimately renal failure. Prevalence of HTN varies in different parts of the world, affecting 28% of adult population in North America, 40% in European countries, 25% in Far East region, 15% in South Asian countries, and 26% in Eastern Mediterranean region [1]. The prevalence and severity varies markedly with age and was expected to raise from 972 million people in the year 2000 to 1.54 billion individuals in 2020. In Pakistan, HTN is regarded as the second commonest deadly disease as there are 12.5 million diagnosed cases of HTN and 12,000 die every year because of complications of this disease. The Pakistan Medical Research Council data published in 1998 revealed that 18% of Pakistani population is hypertensive, but it seems to have much increased since that time, as the prevalence is now 1out of every 3 middle aged persons [2]. 

Despite its high prevalence, identification of its key determinants remains challenging due to its multifactorial and polygenic nature. The genetic, environmental, and dietary factors possibly mediating via hormonal, metabolic, and neurological changes in tissues and organs are manifested as phenotypes [3]; however, the precise mechanism of its occurrence and progression remains obscure. Identification and awareness about prehypertension (pre-HTN) stage with high normal blood pressure (BP) was stressed by 7th JNC report to avoid its progression into established HTN [4].

Genetic studies on Mendelian HTN have provided better understanding of etiological mechanisms including synthesis and degradation of mineralocorticoids and their receptors, renal sodium channels reabsorption mechanisms, regulation of kidney specific sodium-chloride cotransporters, and regulation of renin angiotensin aldosterone system. Thus, information about effects of adrenal hormones aldosterone and cortisol on myocardium, vascular endothelium and HTN is being accumulated [5]. Aldosterone is a mineralocorticoid (MC) hormone, having 30–50% of its total plasma concentration in free form [6]. Cortisol, the glucocorticoid (GC) hormone, on the other hand, has 100-fold higher free levels in circulation than aldosterone with high intrinsic MC activity, though its action is blunted, at the level of kidney, by local conversion to cortisone [7]. Under normal physiological condition, cortisol does not contribute much to MC action in typical target tissues, for example, kidney, but can lead to HTN when this conversion is blunted by deficiency or inhibition of the enzyme 11-beta hydroxysteroid (HSD2) [8]. There is possibility of common sequences of amino acids shared between GC and MC receptors that allow cortisol to bind with the same high affinity as aldosterone binds to MC receptor [9].

The plasma aldosterone secretion is stimulated by either a rise in serum potassium (K^+^) or a fall in serum sodium (Na^+^) concentration. It exerts its effect initially by increasing epithelial Na^+^ channel (ENa^+^C) activity via an aldosterone specific enzyme eventually resulting in increased Na^+^ and decreased K^+^ levels [10]. The phenomenon of aldosterone escape in which the body maintains aldosterone production through mechanisms not involving angiotehsin-II has also been suggested to cause HTN, followed by events like promotion of Na^+^ and water retention, accumulation of extracellular fluid volume, and increased cardiac output. This results in achievement of a steady state with renal K^+^ wasting and increase in arterial BP [11]. Aldosterone also activates MC receptor in vascular smooth muscle, thus decreasing arterial compliance and increasing peripheral resistance [12]. The primary aldosteronism, previously thought to be present only in 1% of individuals with HTN, is now suggested to be present in up to 15%–20% of unselected individuals with HTN [13].

Human adipocytes in overweight and obese individuals have been documented to secrete potent MC releasing factor that simulates adrenal aldosterone production, in addition to expressing a complete renin angiotensin system [14]. Angiotensin-II stimulation of aldosterone release is mediated by AT-1 receptors, that in turn is associated with insulin resistance (IR), resulting in several metabolic changes such as hyperglycemia, dyslipidemia, and HTN by accelerating the atherosclerotic process and endothelial dysfunction [15].

Several mechanisms are suggested for body fat mass influence on secretion of aldosterone and cortisol [16]. These include direct effect of cortisol by increasing hepatic production of angiotensinogen, by binding MC receptor, and increasing vascular reactivity. The elevated levels of free fatty acids activate a neuroendocrine reflex, leading to increased circulating levels of cortisol [17] known to be associated with many dysfunctions including HTN [18].

The prevalence of HTN in Pakistan is one of the highest in the world and it is regarded as a high cardiovascular risk population due to increased incidences of stroke, myocardial infarctions, and end stage kidney disease at younger ages (<45 years) [19]. The purpose of this study was to determine the association of aldosterone and cortisol with anthropometric measures like body mass index and waist circumference and metabolic factors such as fasting blood glucose, total cholesterol, low density lipoproteins, and electrolytes like sodium and potassium in different stages of HTN. It was aimed to identify the key risk factor that triggers the conversion of normal BP into high normal values, which later on leads to progression of pre-HTN stage to HTN stage I. It was presumed to assist in early identification of susceptible individuals for preventive measures.

## 2. Materials and Methods

**Study Design**

Case-control study.

**Sampling**

Convenient.

**Ethical Consideration**

The study was approved by Board of Advanced Studies and Research, University of Karachi.

**Subjects**

The subjects (*n* = 276) were classified into four groups according to cut-off values recommended by 7th JNC report [4] see Table 5.

The age-matched control subjects were selected from general population with normal blood pressure readings without any medications. Pre-HTN group comprised all those subjects who were for the first time told that they have higher normal BP.

The hypertensive subjects (age 25–60 years) representing urban population (as data was collected from largest city of country) were selected amongst patients attending five general practitioners clinics. Most of the subjects were educated and belonged to middle and lower middle socioeconomic class; details are published in previous article [20].

### 2.1. Questionnaire

Demographic data and lifestyle behaviors was collected by purpose designed questionnaire, followed by general physical examination.

Written consent of every participant was taken.

### 2.2. Anthropometric Measurements

#### 2.2.1. Measurement of Body Weight and Height

Subjects were weighed without shoes, in their normal clothing, using a digital scale with an accuracy of ±100 grams. Standing body height was measured without shoes to the nearest 0.5 cm by a commercial stadiometer with the shoulders in the relaxed position and arms hanging freely.

 Body mass index (BMI) was calculated from standard formula (Kg/m^2^). The WHO recommended values for Asian population were taken as reference, >23 overweight and >25 obese [21].

#### 2.2.2. Measurement of Waist Circumference (WC)

It was measured in the middle between 12th rib and iliac crest at the level of umbilicus. Normal values for males were <83 and for females <79 [22].

### 2.3. Blood Pressure Measurement

 Subjects were seated in a chair with their back supported and their arms rested at heart level. Measurement was performed with the subject not having ingested coffee or smoked for 30 minutes and after at least five minutes of rest. The first and fifth Korotkoff sounds were recorded by the height of mercury column on sphygmomanometer. Two readings were taken and averaged.

#### 2.3.1. Biochemical Analysis

Fasting venous blood samples were drawn (after 9–12 hours fasting), centrifuged, and analyzed (by commercially available kits) for estimation of fasting blood glucose (FBG), electrolytes (Na^+^ and K^+^), total cholesterol (TC), and low density lipoproteins (LDL). Serum cortisol and aldosterone were measured by ELISA.

### 2.4. Statistical Analysis

Data was analyzed by SPSS version 10. All variables were presented by mean ± SD. ANOVA was performed to compare four study groups and least significance difference (LSD) test was applied to compare pair-wise groups. Test of Pearson's correlation was applied to assess relationship of hormones with variables determined and shown by Scatter plots. Coefficient determination “*r* ^2^” was also calculated by squaring the value of *r*. It expresses the proportion of the variance in one variable that is accounted for or explained by the variance in other variable. However, it does not demonstrate a causal relationship.

## 3. Results 

The subjects (*n* = 276) were almost equal with respect to sex (male: 49.6%; female 50.4%). The mean age of control group was 37.5 ± 8.54, pre-HTN 39.2 ± 7.73, HTN stage 1 46.2 ± 12.1, and 47.5 ± 11.6 in stage II. Among healthy controls, 52 (69.3%) were found to be overweight (BMI > 23) with significantly higher proportions (*P* = 0.023) of overweight individuals in pre-HTN (80%), HTN stage I (90%),and stage II (76.3%) (Table 1).

The mean BMI (kg/m^2^) of control group (24.9 ± 3.77) was significantly less than that of pre-HTN group (26.4 ± 4.47), HTN stage I (28.4 ± 4.53), and stage II (26.6 ± 5.33) groups (*P* < 0.01). Mean WC of both HTN stage I (97.2 ± 11.4) and II (95.4 ± 13.3) was found to be significantly higher (*P* < 0.01) as compared to control (82.2 ± 13.4) and pre-HTN groups (86.9 ± 17.2) (Table 1). BMI has positive correlation with cortisol in both control and pre-HTN groups and with aldosterone in control group, whereas WC have positive correlation with aldosterone in pre-HTN group only (Table 2).

The mean systolic BP in various groups of control, pre-HTN, and HTN were 106.9 ± 10.7, 131.6 ± 7.1, 147.3 ± 8.4, and 171 ± 15.3. The diastolic BP were 85.9 ± 5.8, 93.7 ± 4.3, and 105.9 ± 9.7 respectively, as compared to control 68.4 ± 9.48 (Table 1).

The mean total cholesterol (TC) level was higher in HTN stage 1(191.4 ± 48.4) as compared to 168.7±33.9 in controls (*P* < 0.01), 171.7 ± 32.3 in pre-HTN (*P* < 0.05), and 179.1 ± 32.1 in stage II (Table 1). TC was positively correlated to aldosterone in pre-HTN group (Table 3). The level of LDL was higher in HTN stage 1 (116.4 ± 32.9) as compared to control (106.5 ± 30.7), pre-HTN (105.6 ± 32.7), and HTN stage II (103.7 ± 29.2) (Table 1). LDL was positively correlated to fasting blood glucose and aldosterone in pre-HTN group (Tables 1 and 3).

The mean fasting blood glucose (FBG) level was in normal range in all groups but was highest in pre-HTN group (108.4 ± 38.2) as compared to controls (96.6 ± 38.4), HTN stage I (103.7 ± 32.7), and stage II (101.0 ± 43.6). FBG was correlated to LDL in pre-HTN group.

The mean aldosterone level was the highest in, HTN stage I (12.41 ± 5.72) as compared to control group (9.17 ± 3.49), pre-HTN (8.76 ± 3.31), and stage II HTN (12.05 ± 6.84). The serum cortisol level in four groups was 7.61 ± 4.15, 9.36 ± 3.25, 9.08 ± 3.97, and 9.99 ± 4.85, respectively.

Serum Na^+^ concentration was within normal limits in all groups (control: 141.8 ± 3.24, pre-HTN 142.4 ± 4.4, stage I 142.2 ± 4.3, and stage II 141 ± 4.72). Serum K^+^ level in four groups was also within normal range (Table 2). Both Na^+^ and K^+^ were positively related to cortisol in controls (Table 2).

The coefficient of determination (*r* ^2^) showed that changes in BMI accounts for 39% of variation in cortisol and 48% interdependence between sodium and cortisol, whereas dependence shared between aldosterone and cholesterol was 53% and with fasting blood glucose 23% (Table 4).

## 4. Discussion

This study investigated the possible interaction of aldosterone and cortisol with certain cardiovascular risk factors like obesity (BMI, WC), dyslipidemia (TC, LDL), and glycemic status of blood (FBG) in different stages of HTN. The study was focused on identification of risk factors in pre-HTN stage, that initiate an increase in set point BP, ultimately leading to progression of this stage into established HTN.

Rapid urbanization and migration towards cities have brought significant changes in lifestyle patterns of our population, especially excessive dietary fat and salt intake and lack of physical activity, resulting in storage of surplus fat in subcutaneous and visceral adipose tissue [23] as evidenced by higher BMI and WC of majority of subjects in our study (Table 1). According to free fatty acid (FFA) hypothesis, dietary fat is converted into triglycerides (TG), broken down into fatty acid, and taken up by fat cells where it is converted to TG again [24]. Body fat accumulation and hyperlipidemia enhance insulin resistance (IR), by increasing free fatty acid in portal blood vessels, impairing insulin signaling on one hand, and by inhibiting key signaling proteins, indicating disturbances in glucose and lipid metabolism (as revealed by significant positive correlation of LDL with FBG in this study), and lead to cortisol secretion on the other hand [25].

**Figure 1:**
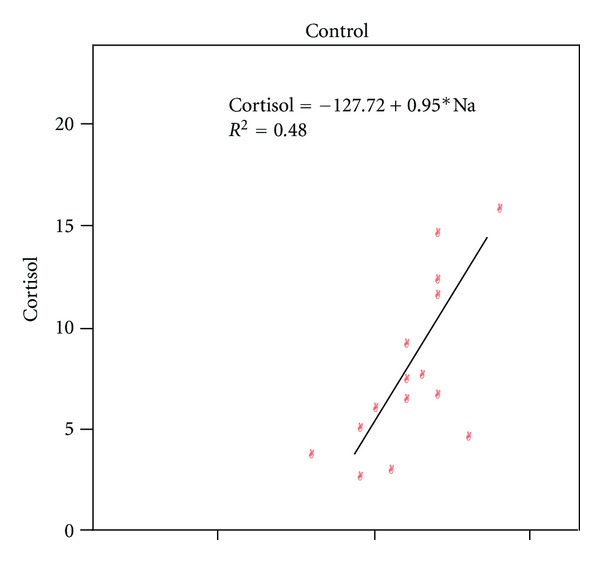
Correlation between sodium (Na^+^) and cortisol.

**Figure 2:**
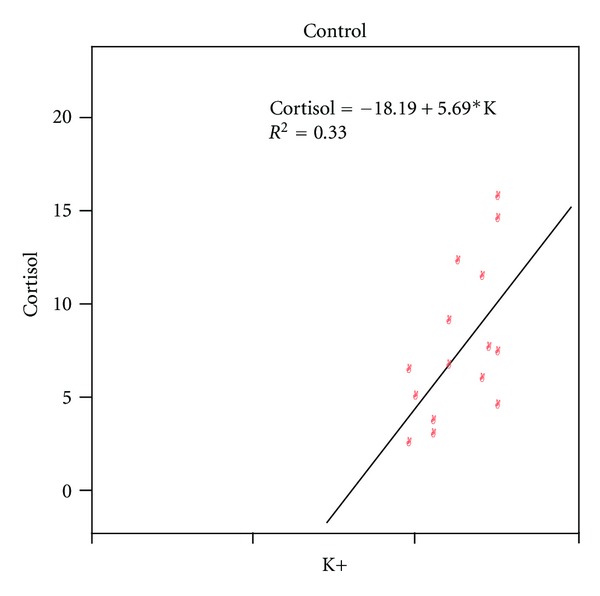
Correlation between potassium (K^+^) and cortisol.

The enzyme hydroxysteroid dehydrogenase type-I (HDS1) converts cortisone to cortisol within insulin target tissue, such as liver, visceral adipose tissue and skeletal muscle, resulting in local GC action which by promoting adipogenesis [26] leads to IR and subsequently hyperinsulinemia; this in turn along with other adipokines results in increased aldosterone production, Na^+^ retention, and HTN [27]. The mean cortisol levels among four study groups were within lower normal range; however, statistical analysis indicated that cortisol level was significantly correlated to BMI, Na^+^, and K^+^ in controls (Figures 1 and 2) and to BMI and WC in pre-HTN group, indicating its association with obesity (Table 3). The relationship between cortisol and obesity has been studied in detail; low circulating cortisol concentrations have been measured in obese individuals, which could be related to increased peripheral metabolism of cortisol [28].

**Figure 3:**
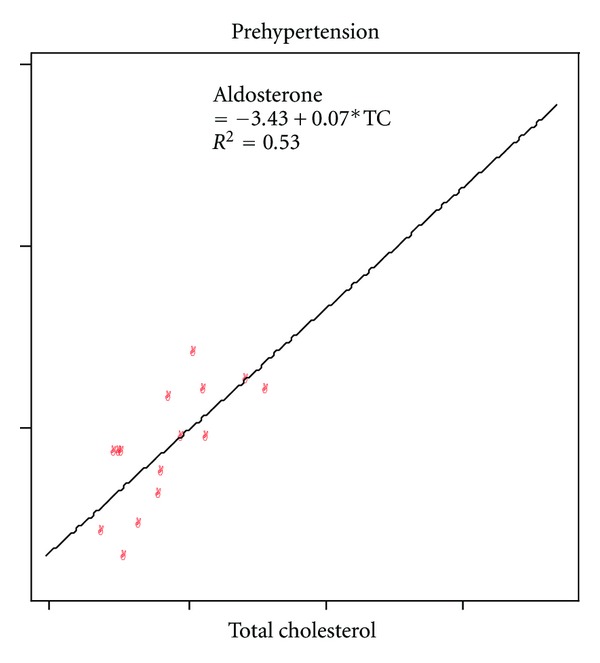
Correlation between total cholesterol and aldosterone.

**Figure 4:**
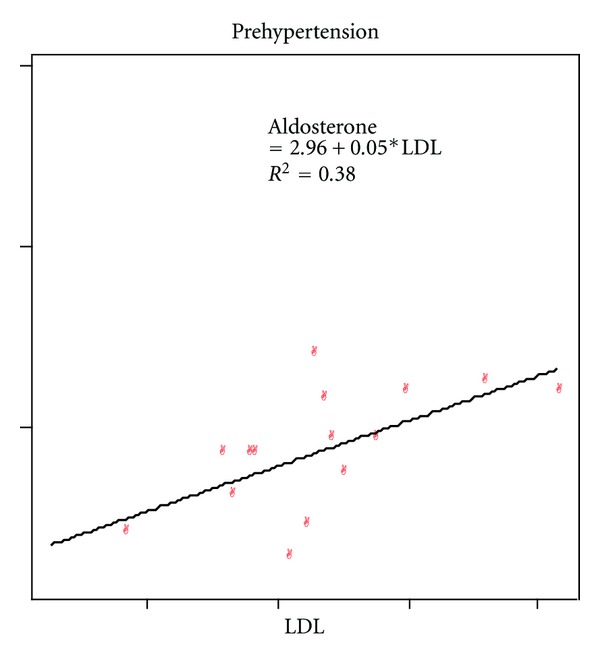
Correlation between low density lipoproteins (LDL) and aldosterone.

The mean aldosterone level in this study was in high normal range and was found to have positive correlation with BMI and WC in controls, and to TC and LDL in pre-HTN group (Figures 3 and 4). Association of aldosterone with obesity, lipid levels, and IR had been confirmed by studies as adipokines and insulin stimulates aldosterone production, which in turn causes fluid retention, endothelial cell dysfunction, atherosclerosis, and HTN [4, 29].

A community-based study reported that even increased plasma aldosterone concentration within physiological range predisposed to the development of HTN [30]. However long-term increase reflects the interaction of unknown genetic and known environmental factors (increased dietary fat and salt intake, decreased physical activity, stress, caffeine consumption, and so forth) leading to eventual phenotype of aldosterone associated HTN and cardiovascular (CV) damage in middle age and beyond. In view of important role played by aldosterone, it is now under consideration that it should be included as the primary screening target for preventing CV events [31]. 

Plasma Na^+^ and K^+^ are major determinants for the mechanical stiffness of endothelial cells. High plasma Na^+^ levels stiffen endothelial cells and block nitric oxide (NO) synthesis (aldosterone is a prerequisite for this condition), whereas high plasma K^+^ levels soften endothelial cells and activate NO release [32]. Clinical studies have documented that a diet low in K^+^ (10–16 mmol/day) coupled with usual Na^+^ intake (120–200 mmol/d) caused Na retention and an elevation of BP; on average systolic BP increased by 6 mmHg and diastolic BP by 4 mmHg in normotensive subjects and by 7 and 6 mmHg, respectively, in hypertensive subjects [33].

Data analysis in this study indicated that Na^+^ level was normal in all four groups; however, it was positively related to BMI and cortisol in controls and to systolic and diastolic BP in pre-HTN group. The strong relationship between Na^+^ and cortisol is very significant, as when in excess cortisol may act as an MC, perhaps by saturating the 11-beta hydroxysteroid-dehydrogenase-2 enzyme that inactivates cortisol at the renal tubules [28]. A study supported that abnormal Na^+^ metabolism at the cellular level may play a role in biochemical pathway leading to HTN [34] and another study confirmed, on epidemiological grounds, the positive link of Na^+^ and a negative link of K^+^ to BP within a single population [35].

The serum K^+^ level was within normal range and was positively related to cortisol in controls which is a significant finding, as high level of K^+^ is considered to be a stimulus for aldosterone secretion. Cortisol and aldosterone bind to the MC receptor with equal affinity, but normal circulatory concentration of cortisol is 100- to 1000-fold higher than those of aldosterone. If 11-beta hydroxysteroid (HSD2) is oversaturated or defective, more cortisol will be available to bind MCR [9, 30], a condition termed as apparent MC excess (AME), characterized by low renin and aldosterone levels, normal plasma cortisol level, and hypokalemia. However because cardiomyocytes lack 11-beta HSD-2, MC receptors are normally occupied by cortisol in a tonic inhibitory fashion and their activation can be triggered by hypoxia, inflammation, and generation of reactive oxygen species causing myocardial damage [36].

Abnormal K^+^ homeostasis is said to accompany many secondary forms as well as uncommon, inherited monogenic forms of HTN, as evidenced by 14% nonhypertensive subjects in Framingham heart study who developed HTN, when followed up to four years; however, other studies found no correlation between serum K^+^ and HTN [37]. In IR, due to a decrease in responsiveness of cells to insulin, K^+^ entry into the cells decreases, and its plasma level increases. The plasma K^+^ level need to increase only 1 meq/L to stimulate aldosterone secretion.

The visceral fat accumulation in upper abdomen also activates sympathetic nervous system and renin angiotensin system. When aldosterone levels are in low- to normal-range and Na^+^ status is unremarkable, cortisol appears to be responsible for MCR activation and aldosterone actions. Increased levels of these hormones in hypertensive subjects may be related to ACTH, which not only increases production of cortisol but also of aldosterone for short-term period [38]. Individuals with less efficient cortisol synthesis maintains a slightly enhanced ACTH drive to adrenal which in long term is likely to cause hyperplasia of both zona fasciculata and glomerulosa, resulting in increased synthetic capacity for both cortisol and aldosterone [39].

## 5. Conclusion

This study concludes the identification of pre-HTN stage among Pakistani population, with successive increase in various risk factors of HTN (obesity, dyslipidemia, hyperglycemia, and Na^+^), in relation to aldosterone and cortisol in different stages of HTN. This study suggests that the interaction of these risk factors with endogenous levels of aldosterone and cortisol may disrupt the set-point BP and result in increased proportion of HTN. 

## 6. Recommendations

Timely and early detection of pre-HTN stage with a few preliminary investigations is an effective method of prevention of cardiovascular disease. The physicians are recommended to actively target lifestyle patterns for multiple risk reduction in these patients, as pre-HTN stage serves as an early warning sign for both patients and clinicians that metabolic changes which ultimately lead to CVD may well be underway. Moreover, aldosterone should be included in primary screening tests for evaluation of hypertensive patients.

## Figures and Tables

**Table 1:** Mean values of different variables (mean ± SD) in controls and stages of hypertension.

Variable/group	Control (*n* = 75)	Pre-HTN (*n* = 55)	HTN stage 1 (*n* = 70)	HTN stage II (*n* = 76)
Age	37.5 ± 8.54	39.2 ± 7.73	46.2 ± 12.1^∗^	47.5 ± 11.6^∗^
Systolic BP	106.9 ± 10.7	131.6 ± 7.1	147.3 ± 8.4	171 ± 15.3
Diastolic BP	68.4 ± 9.48	85.9 ± 5.8	93.7 ± 4.3	105.9 ± 9.7
Body mass index	24.9 ± 3.77	26.4 ± 4.47	28.4 ± 4.53^∗^	26.6 ± 5.33
Waist circumference	82.2 ± 13.4	86.9 ± 17.2	97.2 ± 11.4^∗^	95.4 ± 13.3^∗^
Fasting blood glucose (FBG)	96.6 ± 38.4	108.4 ± 38.2^∗^	103.7 ± 32.7	101 ± 43.6
Total cholesterol (TC)	168.7 ± 33.9	171.7 ± 32.3	191.4 ± 48.4^∗^	179.1 ± 32.1
Low density lipoproteins	106.5 ± 30.7	105.6 ± 32.6	116.4 ± 32.4^∗^	103.7 ± 29.2
Sodium (Na^+^)	141.5 ± 3.24	142.4 ± 4.4	142.2 ± 4.3	141.03 ± 4.72
Potassium (K^+^)	4.5 ± 0.43	4.47 ± 0.42	4.26 ± 0.51	4.41 ± 0.45
Aldosterone	9.17 ± 3.49	8.76 ± 3.31	12.41 ± 5.72	12.05 ± 6.84
Cortisol	7.61 ± 4.15	9.36 ± 3.25	9.08 ± 3.97	9.99 ± 4.85

*The mean difference is significant at the 0.05 level, when compared among four groups.

**Table 2:** Coefficient correlation (*r*) to identify the association of different variables with one another among four groups.

Variable	Aldosterone	Cortisol
BMI		
Control	0.578^∗^	0.538^∗^
Pre-HTN	0.303	0.628^∗^
HTN-I	0.144	0.299
HTN-II	0.544^∗^	0.361
WC		
Control	0.446	0.352
Pre-HTN	0.679^∗∗^	0.263
HTN-I	0.161	0.149
HYN-II	0.345	0.445
FBG		
Control	0.451	0.185
Pre-HTN	0.483	−0.18
HTN-1	0.362	−0.345
HTN-II	−0.102	−0.264
TC		
Control	0.041	0.286
Pre-HTN	0.726^∗∗^	0.308
HTN-I	0.309	0.043
HTN-II	0.128	0.036
LDL		
Control	0.048	0.371
Pre-HTN	0.620^∗^	0.275
HTN-I	0.161	0.208
HTN-II	0.071	0.179
Na^+^		
Control	0.108	0.690^∗∗^
Pre-HTN	0.230	0.292
HTN-I	0.392	0.200
HTN-II	0.259	0.083
K^+^		
Control	0.146	0.578^∗^
Pre-HTN	0.473	0.027
HTN-I	0.439	0.151
HTN-II	0.254	0.045

*Significant at the 0.05 level.

**Significant at 0.01 level.

**Table 3:** Pearson's correlation (*r*) among important variables, showing relationship of different variables with one another in prehypertension group.

Variables	BMI	WC	SBP	DBP	Na^+^	FBG	TC	LDL	Aldost	Cortisol
BMI	1.00	.825^∗∗^	−.274^∗^	−.165	−.126	.207	.078	.201	.303	.628^∗^
WC	.825^∗∗^	1.00	−.356^∗^	−.353^∗^	−.056	.241	−.058	.103	−.263	.679^∗∗^
SBP	−.274^∗^	−.356	1.00	.270^∗^	.328^∗^	−.205	−.093	−.10	.060	−.38
DBP	−.165	−.353	.270^∗^	1.00	.059	−.300	.069	.076	.216	−.50
Na^+^	−.126	−.056	.328^∗^	.059^∗^	1.00	−.267	.034	−.04	.230	.292
FBG	.207	.241	−.205	−.300	−.267	1.00	.205	.289^∗^	.483	−.18
TC	.078	−.058	−.093	.069	.034	.205	1.00	.822^∗∗^	.726^∗∗^	.308
LDL	.201	.103	−.097	.076	−.04	.289^∗^	.822^∗∗^	1.00	.620^∗^	.275
Aldost	.303	−.263	.060	.216	.230	.483	.726^∗∗^	.620^∗^	1.00	.00
Cortisol	.628^∗^	.679^∗∗^	−.380	−.500	.292	−.176	.308	.275	−.005	1.00

*Significant at 0.05 level.

**Significant at 0.01 level.

SBP: systolic blood pressure, DBP: diastolic blood pressure, FBG: fasting blood glucose, TC: total cholesterol, LDL: low density lipoproteins, Aldost: aldosterone.

**Table 4:** Measure of coefficient determination (*r* ^2^) showing the proportion of variance shared among two variables in pre-HTN group.

Variable	Cortisol	Aldosterone
Diastolic BP	25%	—
BMI	39%	—
Cholesterol	—	53%
LDL	—	38%
Fasting blood glucose	—	23%
Serum Na^+^	48%	—
Serum K^+^	33%	22%

**Table 5:** 

Stages	Systolic BP mmHg	Diastolic BP mmHg
Control (*n* = 75)	<120	<80
Prehypertensive (*n* = 55)	>120 and <140	>80 and <90
Hypertension stage I (70)	>140 and <160	>90 and <100
Hypertension stage II (76)	>160	>100
